# Surgical interventions in velopharyngeal dysfunction: comparative perceptual speech and nasometric outcomes for three techniques

**DOI:** 10.1186/s40463-021-00548-4

**Published:** 2022-02-04

**Authors:** Ryan Instrum, Agnieszka Dzioba, Anne Dworschak-Stokan, Murad Husein

**Affiliations:** 1 Department of Otolaryngology–Head and Neck Surgery, Children’s Hospital, London Health Sciences Centre, Victoria Hospital, Schulich School of Medicine and Dentistry, University of Western Ontario, 800 Commissioners Road East, London, ON N6A 5W9 Canada; 2 Thames Valley Children’s Centre, London, ON Canada

**Keywords:** Velopharyngeal dysfunction, Pharyngeal flap, Furlow palatoplasty, Cleft palate, Speech, Hypernasality

## Abstract

**Background:**

The aim of this study was to evaluate speech outcomes following surgical intervention for velopharyngeal dysfunction (VPD). Perceptual speech outcome data were subsequently analyzed in conjunction with patient factors such as congenital abnormalities, presence of cleft lip and/or palate, and age of repair. We hope to aid in the eventual creation of treatment algorithms for VPD, allowing practitioners to tailor surgical technique selection to patient factors.

**Methods:**

A retrospective analysis was performed for all patients who underwent surgical correction of VPD at London Health Sciences Centre between the years 2005 and 2018. Two hundred and two consecutive VPD patients (median age 10.6 years) were followed for an average of 20.2 months after having undergone a superiorly based pharyngeal flap (121), Furlow palatoplasty (72), or sphincteroplasty (9). Speech outcomes were measured via the American Cleft Palate-Craniofacial Association (ACPA) perceptual speech assessment, and MacKay-Kummer Simplified Nasometric Assessment Procedures Revised (SNAP-R) was used to measure nasalence. Comparisons of mean preoperative and postoperative outcomes were made, as well as analyses regarding surgical procedure, syndrome, cleft status, and age.

**Results:**

Mean perceptual scores improved significantly postoperatively (p < .0001), and successful perceptual resonance was identified in 86.1% patients (n = 174). Postoperative perceptual speech scores for three ACPA domains were superior with pharyngeal flap compared to both Furlow palatoplasty and sphincteroplasty ([hypernasality: p < .001, p < .02], [audible nasal emissions: p < .002, p < .05], [velopharyngeal function: p < .001, p < .05]). Success rate was higher in pharyngeal flap (94.2%) than in Furlow palatoplasty (75.0%, p < .001) or sphincter pharyngoplasty (66.7%, p < .001). No significant difference was identified in success rate based on syndrome or cleft status.

**Conclusion:**

Operative management of VPD is highly effective in improving perceptual speech outcomes. Given proper patient selection, all three procedures are viable treatment options for VPD. For those patients identified as appropriate to undergo a pharyngeal flap, robust improvements in speech outcomes were observed.

**Graphical abstract:**

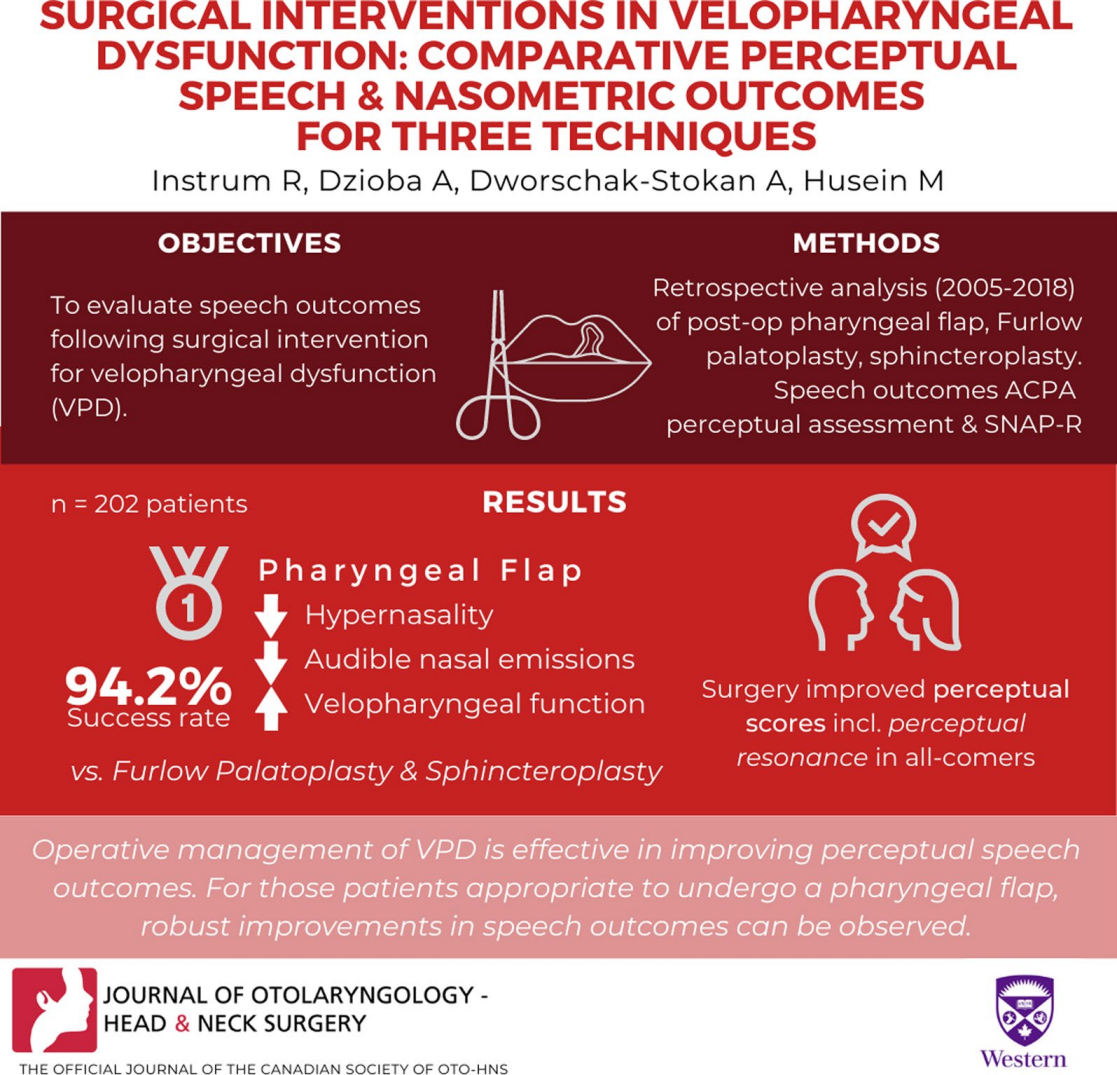

## Background

The velopharyngeal valve is comprised of the soft palate (velum) anteriorly, the lateral pharyngeal walls, and the posterior pharyngeal wall. Speech production relies heavily on the appropriate closure of this valve as the preponderance of oral speech phonemes are created with a sealed port [1]. Closure is primarily achieved via the elevation and retraction of the velum with supplemental contraction of the posterior and lateral pharyngeal walls which serves to functionally separate the nasal passage from the oral cavity and respiratory tract [2].

Velopharyngeal dysfunction (VPD) occurs when this mechanism is disrupted so as to prevent complete valve closure. The etiology of dysfunction may be inadequate tissue volume or mechanical restriction (velopharyngeal insufficiency), neuromuscular deficits (velopharyngeal incompetence), or learned maladaptive articulation (velopharyngeal mislearning) [2–7]. Aberrant speech production results from dysfunction with characteristic hypernasality, nasal air emissions, and diminished vocal intensity [8]. This yields poorly intelligible speech which can have profound implications on quality of life [9].

Treatment strategies for VPD aim to obviate the anatomic gap in the velopharyngeal valve to allow for normal speech resonance [10–16]. Conservative management can take the form of speech therapy, although this is typically reserved for patients with the mislearning subtype or as an adjunct to other modalities [17]. Moreover, prosthetic appliances can be a suitable option for poor surgical candidates and foster modest improvements, although they require a high level of compliance and are not definitive treatment [4, 12, 13, 18]. Surgical intervention remains the mainstay of VPD management insofar as it is capable of definitively correcting the causative structural anomalies while relying less heavily on continual patient compliance [10–16, 19].

Numerous operative techniques exist which have evolved to include modifications and combinations of procedures. The most common techniques include the pharyngeal flap, Furlow palatoplasty (double opposing Z-plasty), and sphincter pharyngoplasty, each with multiple modified versions [10, 11, 15, 16, 20–32]. Procedure selection is partially based on velopharyngeal closure pattern but is also reliant on factors relating to the surgeon such as training, comfort with each technique, and institutional preference [10–12, 15, 16, 22].

## Materials and methods

### Study objectives

Our aim is to present patient data regarding surgical correction of VPD at our center. A comparative analysis of procedure-specific perceptual speech and nasometric outcomes will be performed. Furthermore, treatment outcomes will be correlated with patient-specific considerations such as cleft status, congenital abnormalities, and age. We hope to aid in the creation of treatment algorithms for VPD, thus allowing practitioners to tailor surgical technique selection to patient factors.

### Design

A retrospective analysis was performed on prospectively gathered data from patients who underwent surgical correction of VPD at the Children’s Hospital, London Health Sciences Centre, an academic tertiary care hospital network. All patients on whom the senior author (M.H.) operated between the years 2005 and 2018 (inclusive) were included in the study. Patients were assessed pre and postoperatively by a multidisciplinary team specializing in VPD and cleft palate, and each received concurrent speech therapy throughout. Evaluation included perceptual speech and objective nasometric assessment performed by the same speech-language pathologist (A.D.-S.), as well as functional anatomic examination with flexible nasendoscopy. Speech outcomes from three surgical techniques are described and subgroup analyses have been performed based on characteristics such as presence of cleft/cleft type, comorbid syndromes, and patient age.

Patients undergoing surgery were seen for follow-up by the surgical team 4 weeks after their procedure. Thereafter, patients were evaluated in the VPD clinic with perceptual speech assessment being performed by speech language pathology between 4 and 6 weeks post-operatively as well as at regular intervals during longitudinal follow-up visits. For the purposes of this study, the most recent perceptual speech data for each patient were included in our analysis.

### Procedure selection and technique

For each patient, operative procedure was determined primarily based on closure pattern and etiology of dysfunction. Pharyngeal flap was selected for sagittal and circular closures, poor palate movement yielding central gaps, and large gaps in those with good lateral pharyngeal wall motion. This phenotype is commonly seen following previous cleft palate repair. Furlow palatoplasty was chosen for patients undergoing primary cleft repair or secondary repair, as well as patients with submucous cleft palate. Furlow palatoplasty was also utilized when there was evidence of a muscular diastasis in patients that had previously undergone a palate repair in infancy. This technique allows for lengthening of the palate in patients with a small gap and anatomic replacement of the levator musculature. Sphincter pharyngoplasty was utilized in patients with poor wall movement in all vectors with a resulting coronal closure pattern or in patients with a bow-tie pattern that had central closure but gaps on the lateral aspects of the velopharyngeal ports.

At our center, we perform endoscopically assisted superiorly based pharyngeal flaps with lateral port control as per Hogan, Cable, and Canady [33–35]. Double opposing Z-plasty and sphincter pharyngoplasty technique is similar to that described by Furlow and Orticochea, respectively [36–38].

### Primary outcome

Our primary outcome measure was the American Cleft Palate-Craniofacial Association (ACPA) Perceptual Speech Assessment. Evaluations were made utilizing eight variables from the ACPA clinical database and were performed by one speech-language pathologist specializing in VPD and cleft palate (A.D-S). The variables assessed include: hypernasality, hyponasality, audible nasal emissions, velopharyngeal function, articulation proficiency, overall intelligibility, compensatory articulation, and voice quality. Measurements for six variables were made via a 6-point ordinal scale wherein a score of 1 indicated normal perceptual speech, and a score of 6 specified severe dysfunction. Velopharyngeal function was scored based on a 3-point scale ranging from a score of 1 (adequate) to 3 (inadequate). Compensatory articulation scores were descriptive, with a score of 1 representing no compensatory articulation (i.e. normal) and scores 2–6 each indicating a subtype of compensatory behaviors rather than being indicative of severity.

Comparative analyses were subsequently performed for preoperative and postoperative data in each of the eight variables. A distributive appraisal was then made regarding outcome success following operative intervention. Success was defined as a score of 1 or 2 in hypernasality and audible nasal emission perceptual speech variables [22].

### Statistical analysis of primary outcome

The seven ordinal ACPA categories that were employed for perceptual speech assessment necessitate nonparametric statistical analysis. As such, the Wilcoxon test was used for each of the ordinal speech scales to compare preoperative and postoperative perceptual speech outcomes for all patients. Comparative analysis of ACPA outcomes between the three procedures was made via the Kruskal–Wallis test. Overall operative success, as well as comparative success for each technique, was determined by way of McNemar’s test.

Further subgroup analyses were subsequently performed based on patient traits including patient age, cleft status, and syndrome, as well as combinations therein. Age-related associations were assessed using the Spearman rank correlation. Patients with an identifiable syndrome or genetic abnormality (Table 1) were combined as ‘syndromic’ to facilitate statistical calculations. Comparisons were then made based on the presence or absence of syndrome via the Mann–Whitney U test. The Kruskal–Wallis test allowed for calculations based on cleft status with patients being clustered into non-cleft, cleft, and submucous cleft groups. Furthermore, the Mann–Whitney U test was then employed to evaluate subgroups with isolated syndromes, syndrome and cleft, non-syndrome and non-cleft, and isolated cleft. The Holm’s sequential Bonferroni correction was applied to the p-values to account for the multiple statistical tests that were undertaken in the present study; the adjustment reduces the risk of committing a Type I error, while increasing statistical power [39]. The Holm’s sequential procedure is a stepwise modification of the traditional Bonferroni procedure that ranks tests of significance in ascending order of p-values and alters the magnitude of adjustment as a function of the p-value order. Adjusted p-values < 0.05 were considered statistically significant.

**Table 1:** Population data

	n (%)
Patients (total)	202
*Gender*	
Female	84 (41.6%)
Male	118 (58.4%)
Median age at time of surgery	10.6 years
Mean age at time of surgery	12.4 years
*Mean postoperative time*	
Perceptual assessment	20.2 mo
Nasometry	15.2 mo
*Procedure*	
Pharyngeal flap	121 (59.9%)
Furlow palatoplasty	72 (35.6%)
Sphincter pharyngoplasty	9 (4.5%)
*Cleft status*	
Cleft	101 (50.0%)
SMCP	44 (21.8%)
Noncleft	57 (28.2%)
*Syndromes*	
Syndromic	59 (29.2%)
VCFS/Di George	16 (7.9%)
Pierre Robin sequence	13 (6.4%)
Neurofibromatosis Type 1	5 (2.5%)
Kleinfelter syndrome	5 (2.0%)
Syndrome NYD	7 (3.5%)
Other syndromes	14 (6.9%)
*Cleft/syndrome subgroups*	
Noncleft, nonsyndromic	28 (13.9%)
Isolated syndrome	29 (14.4%)
Isolated cleft	115 (56.9%)
Cleft, syndromic	30 (14.9%)

### Secondary outcomes

Nasalance was our secondary outcome, and this was quantified utilizing nasometry (Kay Pentax Nasometer II). Measurements were made using the Picture-Cued Subtest of the MacKay-Kummer Simplified Nasometric Assessment Procedures Revised (SNAP-R) which consists of four oral consonants and one nasal phoneme [40, 41]. The use of a nasal phoneme served to surveille for potential overcorrection postoperatively. Results of the four oral consonants were averaged for each patient, and statistical evaluations were performed on preoperative and postoperative timepoints for all patients, as well as comparative analyses using the subgroups outlined in the primary outcome methods. Additionally, determinations of outcome success were made. Success using the SNAP-R is defined as average oral scores within 3 standard deviations (SD) from the normative mean, which approximately corresponds to nasalance scores less than 24 [41]. Importantly, oral SNAP-R data are not normally distributed, and no consistent cut-off has been established in the literature. Publications on the tool and its application describe 2 SD above the mean as representing the beginning of a borderline region wherein many patients with normal overall resonance will exist [41, 42]. Scores in the nasal phoneme were considered successful if they were within 1 SD of the mean (< 64) [22].

### Statistical analysis of secondary outcome

Parametric statistical analyses were implemented for nasalance data; SNAP-R scores were determined to be normally distributed based on visual inspection of histograms and Shapiro–Wilk’s test of normality. Comparative assessments of preoperative and postoperative timepoints were made with paired t-tests, and independent samples t-tests were employed to evaluate mean SNAP-R scores between the two timepoints. McNemar’s test was used to determine outcome success, as defined in the preceding section. Furthermore, subsequent subgroup analyses were carried out in a similar fashion to those outlined in the primary outcome statistical methods. Comparisons of mean SNAP-R score at each timepoint, as well as overall score change between them, were made based on procedure and subgroup via analysis of variance (ANOVA) and independent-samples t-tests, respectively. Spearman rank correlation was again used for associations based on age. As per above, the Holm’s sequential Bonferroni correction was applied to the p-values and adjusted p-values of < 0.05 were considered statistically significant.

## Results

### Population data

Two hundred and six consecutive patients underwent operative intervention for VPD over the study interval. Speech assessment data for both pre-operative and post-operative timepoints were available for 202 patients included in the present study (4 patients excluded for missing speech data and loss to follow-up). Mean age at the time of surgery was 12.4 years (SD = 8.8) with a median age of 10.6 years, and ages ranged from 1.6 to 69.0 years. Patients were followed for an average of 20.2 months. Three surgical procedures were utilized: superiorly based pharyngeal flap (59.9%, n = 121), Furlow palatoplasty (35.6%, n = 72), and sphincter pharyngoplasty (4.5%, n = 9). General population and subgroup date are summarized in Table 1. Fifty-nine patients (29.2%) had an identifiable syndrome or genetic abnormality. Twenty-eight subjects (13.9%) had neither a cleft nor syndrome. Clefts were identified in 101 patients (50%) and submucous clefts were present in 44 (21.8%).

### Primary outcome

#### ACPA perceptual speech scores

Combined ACPA speech outcome data for all patients at preoperative and postoperative timepoints are represented in Table 2. Overall, there was found to be a statistically significant improvement in hypernasality, audible nasal emission, velopharyngeal function, articulation proficiency, and overall intelligibility. When categorized by operative procedure (Table 3), all three surgical procedure groups experienced significant improvements in the same domains as the aforementioned combined group.

**Table 2:** Mean ACPA speech outcomes, combined

ACPA speech variable	Pre-op (SD)	Post-op (SD)	p value
Hypernasality	4.30 (1.07)	1.68 (1.02)	< .0001
Hyponasality	1.19 (0.47)	1.08 (0.28)	< 0.005
Audible nasal emissions	4.12 (1.18)	1.72 (1.04)	< .0001
Velopharyngeal function	2.95 (0.24)	1.44 (0.72)	< .0001
Articulation proficiency	2.88 (1.53)	1.94 (1.19)	< .0001
Overall intelligibility	3.33 (1.30)	1.93 (1.14)	< .0001
Voice quality	1.21 (0.65)	1.15 (0.54)	NS

*ACPA* American Cleft Palate-Craniofacial Association, *NS* not significant

p ≤ 0.05

**Table 3:** Mean ACPA speech outcomes by procedure

ACPA speech variable	Pre-op (SD)	Post-op (SD)	p value
*Pharyngeal flap*			
Hypernasality	4.32 (1.02)	1.40 (0.70)	< .0001
Hyponasality	1.21 (0.47)	1.08 (0.28)	< 0.005
Audible nasal emmisions	4.13 (1.14)	1.45 (0.68)	< .0001
Velopharyngeal function	2.98 (0.20)	1.25 (0.52)	< .0001
Articulation proficiency	2.72 (1.43)	1.82 (1.05)	< .0001
Overall intelligibility	3.28 (1.16)	1.75 (0.99)	< .0001
Voice quality	1.20 (0.68)	1.13 (0.52)	NS
*Furlow palatoplasty*			
Hypernasality	4.15 (1.11)	2.01 (1.20)	< .0001
Hyponasality	1.17 (0.48)	1.08 (0.28)	< 0.01
Audible nasal emmisions	4.06 (1.23)	2.13 (1.33)	< .0001
Velopharyngeal function	2.92 (0.28)	1.71 (0.86)	< .0001
Articulation proficiency	3.10 (1.64)	2.13 (1.42)	< .0001
Overall intelligibility	3.35 (1.46)	2.19 (1.33)	< .0001
Voice quality	1.22 (0.61)	1.15 (0.57)	NS
*Sphincter pharyngoplasty*			
Hypernasality	5.11 (1.05)	2.67 (1.58)	< .0001
Hyponasality	1.11 (0.33)	1.11 (0.33)	NS
Audible nasal emmisions	4.56 (1.24)	2.22 (1.20)	< .0001
Velopharyngeal function	2.89 (0.33)	1.89 (0.93)	< .0001
Articulation proficiency	3.33 (1.80)	2.11 (0.78)	< .0001
Overall intelligibility	3.78 (1.72)	2.11 (0.93)	< .0001
Voice quality	1.33 (0.50)	1.33 (0.50)	NS

*ACPA* American Cleft Palate-Craniofacial Association, *NS* not significant

p ≤ 0.05

Pharyngeal flap postoperative ACPA scores were statistically superior (i.e. lower value) than both Furlow palatoplasty and sphincter pharyngoplasty in the following four domains: hypernasality (p < 0.001; p < 0.02, respectively), audible nasal emission (p < 0.002; p < 0.05), and velopharyngeal function (p < 0.001; p < 0.05). There were no significant differences identified in the remaining domains or between Furlow and sphincter pharyngoplasty groups. The quantitative ACPA score change (delta) from preoperative to postoperative timepoints was significantly greater in pharyngeal flap patients when compared to patients having undergone Furlow palatoplasty in hypernasality (p < 0.001), and audible nasal emission (p < 0.001). Velopharyngeal function delta was superior following pharyngeal flap than both Furlow and sphincter pharyngoplasty (p < 0.001; p < 0.04, respectively). No significant variation in preoperative ACPA scores between procedures were found.

#### Distribution of successful ACPA outcomes

The overall rate of operative success (i.e. normal perceptual resonance postoperatively) in all patients was 86.1% (n = 174). Success rate by procedure is depicted in Table 4. Pharyngeal flap patients (94.2%) were found to have a superior rate of success than either Furlow (75.0%) or sphincter pharyngoplasty (66.7%). There was a statistically significant difference in success rate across all three groups.

**Table 4:** Distribution of successful speech resonance outcomes

Procedure	Success rate (%)	n	p value
Pharyngeal flap	94.21	114	p < .0001^†^
Furlow palatoplasty	75.00	54	p < .0001
Sphincter pharyngoplasty	66.67	6	p < .0001
Overall	86.14	174	

^†^Significance calculated across all groups

### Primary outcome subgroup analysis

#### Age

A significant inverse correlation was identified between age and preoperative ACPA scores in the domains of articulation proficiency (ρ = -0.392, p < 0.0001) and overall intelligibility (ρ = -0.240, p < 0.01), as younger patients had poorer scores prior to operative management. Furthermore, this translated into larger improvement postoperatively as there was a significant inverse proportionality between age and preoperative to postoperative score change in articulation proficiency (ρ = -0.444, p < 0.0001) and overall intelligibility (ρ = -0.263, p < 0.02).

#### Length of follow-up

Longer follow-up was associated with greater changes in two ACPA variables, as a positive correlation was identified between follow-up time and preoperative to postoperative score change in articulation proficiency (ρ = 0.228, p < 0.002), and overall intelligibility (ρ = 0.243, p < 0.04).

#### Syndromic versus non-syndromic

When speech data were analyzed based on the presence or absence of an identifiable genetic syndrome or anomaly, syndromic patients were found to have poorer preoperative ACPA scores in articulation proficiency (p < 0.05). Postoperative articulation proficiency (p < 0.04) and voice quality (p < 0.05) scores were also inferior in the syndromic group. However, there were no discernible differences in score change from preoperative to postoperative timepoints in any speech variable. Moreover, no significant difference was observed regarding the proportion of successful outcomes for syndromic (83.1%) and non-syndromic (87.4%) patients.

#### Cleft status

Patients were evaluated based on cleft status by placing them in non-cleft, cleft palate, or submucous cleft palate (SMCP) subgroups. No significant differences in ACPA scores were found between cleft status subgroups. Success rates between subgroups were comparable for all cleft statuses, as no significant difference was identified for non-cleft (86.0%), cleft (86.1%), and SMCP (86.4%) resonance outcomes.

#### Syndrome and cleft

Patients were also grouped based on syndrome and cleft status: non-cleft/non-syndromic, isolated cleft, isolated syndrome, cleft/syndrome. No significant differences in speech variable scores were found between syndrome and cleft subgroups. Furthermore, outcome success rate did not vary significantly between the four groups (non-cleft/non-syndromic—0489.3%; isolated cleft—87.0%; isolated syndrome—82.8%; cleft/syndrome—83.3%).

### Secondary outcome

#### Nasalance: SNAP-R scores

Nasalance outcomes are presented in Table 5. Overall, these data show significantly improved mean SNAP-R scores in both oral and nasal phonemes from preoperative to postoperative timepoints.

**Table 5:** Mean SNAP-R scores and distribution of successful nasometric outcomes

Procedure	Pre-op (SD)	Post-op (SD)	p value	n
*Pharyngeal flap*				
SNAP-R Oral Score	46.78 (14.12)	21.30 (12.00)	< .0001	107
SNAP-R Nasal Score	69.18 (9.89)	63.25 (10.76)	< .01	107
*Success rate*				
Oral	7.62%	71.96%		
Nasal	26.67%	50.47%		
*Furlow palatoplasty*				
SNAP-R Oral Score	44.89 (15.56)	27.77 (17.53)	< .0001	59
SNAP-R Nasal Score	67.83 (9.65)	68.56 (9.49)	NS	59
*Success rate*				
Oral	5.66%	57.63%		
Nasal	33.96%	28.81%		
*Sphincter pharyngoplasty*				
SNAP-R Oral Score	54.14 (12.61)	36.78 (16.64)	< .03	9
SNAP-R Nasal Score	73.33 (7.58)	72.00 (4.53)	NS	9
*Success rate*				
Oral	0.00%	22.22%		
Nasal	11.11%	11.11%		
*Overall*				
SNAP-R Oral Score	46.10 (14.58)	24.15 (14.73)	< .0001	175
SNAP-R Nasal Score	68.80 (9.74)	65.22 (10.61)	< .001	175
*Success rate*				
Oral	6.59%	64.57%		
Nasal	28.14%	41.14%		

*SNAP-R* Simplified Nasometric Assessment Procedures Revised

When organized by procedure, no statistically significant variation was identified in pretreatment scores. However, postoperative oral and nasal SNAP-R in the pharyngeal flap group were significantly better than Furlow palatoplasty (oral: p < 0.009, nasal: p < 0.001) or sphincter pharyngoplasty (oral: p < 0.006, nasal: p < 0.04). Pharyngeal flap patients exhibited a greater degree of change between preoperative and postoperative timepoints than Furlow palatoplasty in both nasalance scores (oral: p < 0.005, nasal: p < 0.0001).

#### Distribution of successful nasalance outcomes

Rates of successful outcomes accompany SNAP-R score data in Table 5. Success rate for all the entire cohort improved significantly from preoperative to postoperative timepoints in oral scores (p < 0.0001). Successful oral and nasal nasalance outcome rates were superior in the pharyngeal flap group than both Furlow and sphincter pharyngoplasty groups.

### Secondary outcome subgroup analysis

#### Syndromic versus non-syndromic

No significant difference was detected in pretreatment or postoperative SNAP-R scores. However, the degree of score change in syndromic patients was significantly reduced in oral scores compared to the non-syndromic group (p < 0.05).

#### Cleft status

No significant differences in SNAP-R scores were found based on cleft status.

#### Syndrome and cleft

Oral SNAP-R score changes in the isolated cleft group between pretreatment and posttreatment were greater compared to the isolated syndrome group (p < 0.05).

### Postoperative complications

The only serious postoperative complication observed in our cohort was obstructive sleep apnea which was identified in 3 pharyngeal flap patients (2.48%) postoperatively and required takedown. Otherwise, only minor wound complications (n = 5, 2.48%) were noted.

## Discussion

The data presented represent one of the largest studies published on the topic of surgical intervention for velopharyngeal dysfunction. When compared to reports with long-term follow-up (≥ 12 months) and postoperative perceptual speech assessment using a published scale, this study involves the largest sample size to date [10]. Longitudinal evaluation is essential when considering operative outcomes in this domain as resonance requires approximately one year to stabilize [43–45]. Moreover, insofar as operative success in VPD is measured via functional outcomes, the use of published resonance assessment tools enhances understanding and reproducibility of results.

At our center, procedure selection is largely determined by closure pattern. The dynamics of closure, its configuration, and associated tissue bulk are discerned via endoscopic examination and videofluoroscopy in conjunction with nasometric quantification of airflow during phonation. We choose this combination of 2D imaging in lieu of other radiographic modalities such as 4D-CT as it provides the necessary anatomical information and quantitative measures prior to surgery without high levels of radiation exposure [46].

Based on our results, all three surgical techniques appear to be viable options in the treatment of VPD with the goal of achieving normal speech resonance. These data suggest that the superiorly based pharyngeal flap and Furlow palatoplasty are well-suited to alleviate hypernasality, audible nasal emissions, and compensatory articulations, while improving velopharyngeal function, articulation proficiency, and overall intelligibility. Of note, the Furlow group included patients undergoing either primary cleft repair or secondary repair.

Given the relative paucity of speech data for patients having undergone sphincter pharyngoplasty, it is difficult to draw broad conclusions based on these results. The comparative dearth of patients in this group is multifactorial and is likely due to a combination of a lack of patients with poor wall movement in all axes (the closure pattern by which this procedure is selected at our center), surgeon training and preference. Indeed, a large proportion of our center’s population that undergo VPD corrective surgery had a previous cleft palate repair which is typically treated with a pharyngeal flap due to the palate being the reason for the velopharyngeal dysfunction. However, the patients that were selected for the sphincteroplasty procedure performed reasonably well in terms of postoperative speech resonance. This group also had a larger proportion of syndromic patients and patients undergoing revision repair than the other two groups. Interestingly, the only domain in which preoperative ACPA scores were found to differ significantly between procedures was hypernasality which was found to be worse in sphincter pharyngoplasty when compared to Furlow. It is not clear why this is the case, but one possibility could be that sphincter pharyngoplasty is selected for patients with poor velopharyngeal wall movement in all vectors. Presumably this would imply more severely impaired sphincter competence and lead to a higher proportion of expelled air exiting through the nasal passage. Mean preoperative audible nasal emission scores were higher in this group as well, just not significantly so due to the small sample size.

Patients that underwent pharyngeal flaps were found to have superior outcomes in terms of overall perceptual resonance success rate, as well as quantitatively in nearly all perceptual speech domains. These results may be due to advantages intrinsic to the procedure itself. However, patient selection may play a role in that anatomical and functional elements that determine patient candidacy for pharyngeal flap may be inherently more amenable to surgical intervention. A large proportion of patients in our cohort had repaired cleft palates, and the pharyngeal flap tends to be the workhorse in this population [47]. Furthermore, as mentioned, the relative paucity of sphincter pharyngoplasty data limits the applicability of comparisons made therein. Interestingly, normal speech resonance rates did not vary significantly based on either syndrome or cleft status.

Of note, the average age calculated in our study is higher than what the literature would deem ideal for operative intervention in VPD. This is largely due to the fact that we have elected to include all patients who underwent surgical correction of VPD over our stated interval. These patients include those who have undergone previous repairs as well as outliers who developed VPD in later stages of life due to a variety of circumstances. Patients were scheduled for surgery as soon as was feasible after their original consultation. Similarly, our series contains adults that have come to us for various reasons including post-oncologic surgery on their palate. Subsequent analyses will be made based on other important classifications (e.g. revision surgeries, primary vs. secondary Furlow, SMCP etc.) which we feel deserve to be addressed in a separate manuscript. Moreover, given the sample size of this study, further evaluation can be performed regarding the association of factors such as syndrome subtype, medical comorbidities, age at the time of surgery, requirement of revision, and procedure choice as they relate to functional outcomes and complications. These data, in addition to those presented, will serve to aid in algorithm generation for VPD management.

## Conclusion

Operative management of VPD is highly effective in improving perceptual speech outcomes. Given proper patient selection, all three procedures are viable treatment options for VPD. For those patients identified as appropriate to undergo a pharyngeal flap, robust improvement in speech outcomes were observed.

## Data Availability

The datasets used and/or analyzed during the current study are available from the corresponding author on reasonable request.
